# Multiphysics Optical–Thermal and Mechanical Modeling of a CMOS-SOI-MEMS Infrared Sensor with Metasurface Absorber †

**DOI:** 10.3390/s25226819

**Published:** 2025-11-07

**Authors:** Moshe Avraham, Yael Nemirovsky

**Affiliations:** Electrical and Computer Engineering Department, Technion—Israel Institute of Technology, Haifa 3200003, Israel; smoa@campus.technion.ac.il

**Keywords:** CMOS-SOI, MEMS, infrared sensor, metasurface absorber, multiphysics modeling, FDTD, thermal FEA, uncooled detector, MIM

## Abstract

Infrared (IR) thermal sensors on CMOS-SOI-MEMS platforms enable scalable, low-cost thermal imaging but require optimized optical, thermal, and mechanical performance. This paper presents a multiphysics modeling framework to study the integration of Metasurface absorbers into a Thermal CMOS-SOI-MEMS IR sensor. Using finite-difference time-domain (FDTD) simulations, we demonstrate near-unity absorption at targeted wavelengths (e.g., 4.26 µm for CO_2_ sensing, 10 µm for thermal imaging) compared to conventional absorbers. The absorbed power, calculated from blackbody irradiance, drives thermal finite element analysis (FEA), confirming high thermal isolation and maximized temperature rise (ΔT) while quantifying the thermal time constant’s sensitivity to Metasurface mass. An analytical RC circuit model, validated against 3D FEA, accurately captures thermal dynamics for rapid design iterations. Mechanical modal and harmonic analyses verify structural integrity, with natural frequencies above 20 kHz, ensuring resilience against mechanical resonances and environmental vibrations. This holistic framework quantifies trade-offs between optical efficiency, thermal responsivity, and mechanical stability, providing a predictive tool for designing high-performance, uncooled IR sensors compatible with CMOS processes.

## 1. Introduction

Thermal infrared (IR) sensors are indispensable for a wide range of applications, including night vision, thermal imaging, environmental monitoring, gas sensing, and industrial process control [1,2,3,4]. The demand for compact, high-performance, and cost-effective uncooled IR detectors has spurred significant advancements in Micro-Electro-Mechanical Systems (MEMS) fabricated on CMOS-compatible Silicon-On-Insulator (SOI) platforms. These CMOS-SOI-MEMS platforms offer exceptional thermal isolation, reduced parasitic effects, and seamless integration with on-chip electronics, enabling scalable and low-power sensing solutions [5,6,7]. By leveraging standard CMOS fabrication processes, these platforms pave the way for affordable, high-volume production of IR sensors suitable for both consumer and industrial applications.

The Thermal CMOS-SOI-MEMS (TMOS) sensor represents a breakthrough in uncooled IR detection, utilizing a suspended MOSFET as the sensing element to transduce IR-induced temperature changes into highly sensitive electrical signals [8,9,10,11]. The TMOS architecture achieves superior responsivity by maximizing the temperature rise (ΔT) on a thermally isolated micro-platform, supported by thin holding arms that minimize conductive heat losses. Key to its performance is efficient absorption of incident IR radiation across the 2–14 µm wavelength range, which is critical for applications such as CO_2_ gas sensing (4.26 µm) and thermal imaging (8–14 µm). To address this, advanced nanophotonic components, such as Metasurface absorbers based on metal–insulator–metal (MIM) structures, have emerged as a powerful solution [12,13,14,15]. These Metasurfaces achieve near-unity absorption at targeted wavelengths while remaining fully compatible with CMOS fabrication, offering spectral selectivity for application-specific sensing.

In our previous work [16], we investigated how incorporating MIM absorbers affects the optical and thermal performance of CMOS-SOI-MEMS thermal IR sensors, and we detailed the simulation methodology as well as the influence of MIM geometry on the absorption spectrum.

However, integrating Metasurface absorbers into CMOS-SOI-MEMS sensors introduces complex trade-offs across optical, thermal, and mechanical domains. Enhanced absorption increases thermal capacitance, potentially slowing the sensor’s temporal response, while added mass may alter the mechanical stability of the suspended microstructure. Existing studies often focus on isolated aspects, such as electromagnetic design of absorbers or simplified thermal modeling, failing to capture the coupled interactions that govern overall sensor performance [17,18]. This lack of a holistic multiphysics framework limits the ability to optimize responsivity, speed, and structural integrity simultaneously, hindering the translation of advanced optical concepts into manufacturable devices.

**Figure 1:**
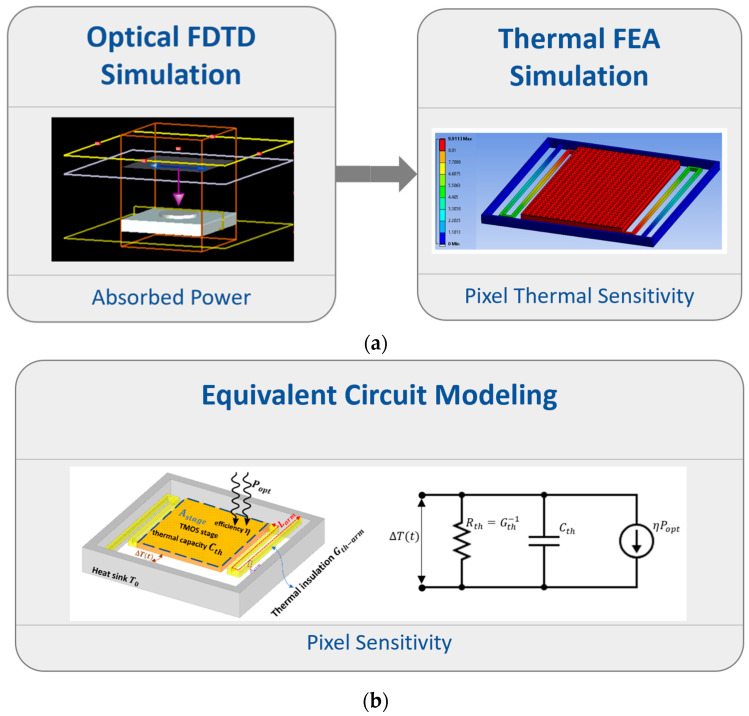
Multiphysics modeling framework for the CMOS-SOI-MEMS thermal IR sensor. (**a**) The sequential coupling of optical absorption efficiency and thermal domains: Optical FDTD Simulation computes the absorbed optical power, which is used as the heat source input for the Thermal FEA Simulation to determine the pixel’s thermal sensitivity. (**b**) Equivalent circuit model illustrating thermal dynamics, where the calculated thermal conductance and thermal capacitance are represented by an RC circuit driven by the theoretically absorbed optical power.

This work addresses this gap by developing a comprehensive multiphysics modeling framework for a TMOS sensor enhanced with Metasurface absorbers. The optical response was modeled using Lumerical FDTD Solutions [19], where finite-difference time-domain (FDTD) simulations quantified the absorption efficiency and incident radiation distribution, serving as input heat sources for the subsequent thermal finite element analysis (FEA). The thermal and mechanical simulations were conducted using ANSYS Mechanical 2022 R2 [20], with the thermal response validated against an analytical lumped-element RC circuit model to evaluate the sensor’s temperature rise and dynamic behavior, revealing the influence of Metasurface mass on the thermal time constant. Furthermore, mechanical modal and harmonic response analyses assessed the impact of Metasurface integration on natural frequencies and vibration modes, ensuring structural robustness under environmental excitations. As illustrated in Figure 1, this integrated optical–thermal–mechanical modeling approach provides a predictive design framework that enables systematic exploration of trade-offs toward high-responsivity, fast, and mechanically stable CMOS-compatible IR sensors for advanced applications.

## 2. Sensor Design and Architecture

### 2.1. TMOS Sensor Architecture

The CMOS-SOI-MEMS infrared sensor employs a TMOS pixel architecture, featuring a nano-machined, suspended transistor fabricated in a standard 0.13-µm CMOS-SOI process for monolithic integration with readout electronics [10]. The core sensing element is a thermally isolated metal-oxide-semiconductor field-effect transistor (MOSFET) operating in the subthreshold regime, which converts absorbed IR radiation into temperature-dependent electrical signals with high sensitivity and low power consumption.

**Figure 2:**
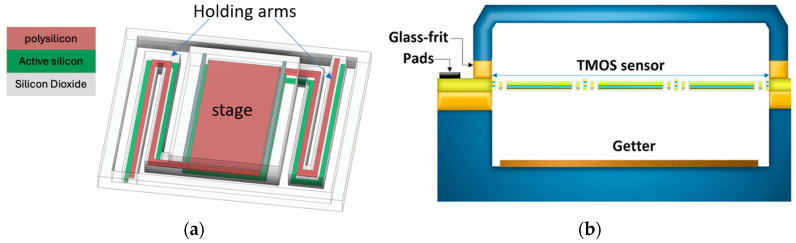
Schematic TMOS sensor (**a**) 3D schematic; (**b**) cross-section of a wafer-level processed (WLP) TMOS sensor array.

The pixel comprises a suspended thermal platform (stage) anchored by thin holding arms, which ensure mechanical stability while minimizing thermal conductance to the substrate. Fabrication involves dry etching techniques, including reactive ion etching (RIE) for front-side dielectrics and deep reactive ion etching (DRIE) for back-side silicon removal, with the buried oxide layer serving as an etch stop. To enhance thermal isolation, the arms incorporate active silicon and polysilicon layers rather than metal interconnects, reducing conductive heat flow. Additional isolation is achieved through wafer-level vacuum packaging (WLP) via glass-frit bonding, incorporating a getter layer to sustain a high vacuum (~1 Pa) and suppress gas conduction and radiative losses [21]. Figure 2 illustrates the TMOS architecture.

### 2.2. Metamaterial Integration

To enhance the optical performance of the TMOS sensor, Metasurface absorbers based on metal–insulator–metal (MIM) structures are integrated atop the suspended stage. Metasurfaces, engineered nanostructures that control electromagnetic wave propagation at subwavelength scales, enable tailored absorption properties unattainable with conventional materials [17,18,22,23]. While this study evaluates two narrowband MIM configurations as exemplars, the proposed multiphysics modeling framework is versatile, applicable to any absorber architecture—whether narrowband, broadband, multilayer, or with varied structural geometries—ensuring broad relevance to diverse IR sensing applications.

The two representative MIM configurations demonstrate specific functionalities:

- Configuration 1: Optimized for peak absorption at 4.26 µm, targeting the CO_2_ absorption band for non-dispersive infrared (NDIR) gas sensing.
- Configuration 2: Designed for narrowband absorption near 10 µm, suitable for biomedical sensing (e.g., protein vibrational fingerprints) and thermal imaging.

**Figure 3:**
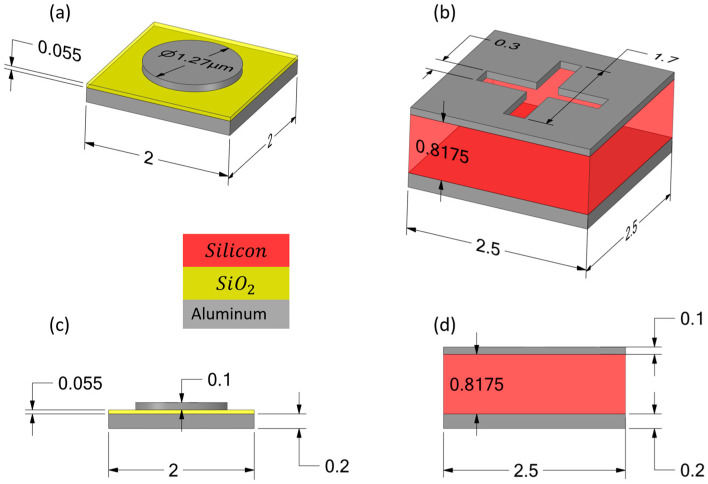
Schematic of the Metasurface absorber unit cell configurations integrated into the TMOS sensor. (**a**) Configuration 1: MIM structure for absorption at 4.26 µm. (**b**) Configuration 2: MIM structure for absorption at 10 µm. (**c**) Side view of Configuration 1. (**d**) Side view of Configuration 2. Layer materials, thicknesses, and unit-cell dimensions are indicated (in µm).

Each MIM unit cell comprises periodic layered stacks, compatible with CMOS post-processing, arranged to form a Metasurface covering the pixel’s active area. The configurations serve to illustrate the framework’s ability to quantify the optical efficiency, thermal dynamics, and mechanical stability of any absorber design. This approach evaluates absorption enhancement, thermal capacitance effects, and mass-induced frequency shifts, as detailed in subsequent sections. Figure 3 depicts the MIM unit cell configurations, including materials, thicknesses, and dimensions (in µm).

It should be noted that the corresponding optical efficiency and absorption results for the MIM 2 structure (shown in Section 3) are presented only for the long-wave infrared (LWIR) range of 8–14 µm. This focus reflects the design intent of the MIM 2 configuration, which was optimized specifically for LWIR operation relevant to multispectral pixel development. Although the full 2–14 µm spectrum was simulated, any additional peaks that may appear in the 2–8 µm range were not emphasized, as external optical filtering is assumed in the intended application. The slightly jagged appearance of the MIM 2 optical efficiency curve in Figure 7 arises from the finite frequency resolution used in the simulation, which affects the smoothness of the plotted response.

## 3. Optical Analysis

The optical performance of the CMOS-SOI-MEMS TMOS sensor hinges on its ability to efficiently absorb incident infrared (IR) radiation, which directly drives the thermal response. This section presents a comprehensive optical modeling framework using (FDTD) simulations to quantify absorption efficiency across the 2–14 µm band. The framework calculates incident irradiance, electromagnetic power dissipation, and absorption characteristics for baseline CMOS, titanium nitride (TiN)-enhanced, and Metasurface-enhanced configurations. While two metal–insulator–metal (MIM) Metasurface designs are evaluated as exemplars, the methodology is versatile, applicable to any absorber architecture—narrowband, broadband, multilayer, or varied geometries. Results provide heat sources for thermal analysis (Section 4).

### 3.1. Incident Irradiance and Radiometric Quantities

To link the electromagnetic simulations with realistic sensor operation, it is necessary to relate blackbody radiation to the optical power absorbed in the detector. The fundamental radiometric quantity is the spectral radiance $L_{\lambda }(T)$, which specifies the emitted power per unit area, per unit solid angle, and per unit wavelength of a blackbody at temperature T [24,25]:

(1) $$L_{\lambda }\left(T\right)=\frac{2hc^{2}}{\lambda^{5}\exp\left(\frac{hc}{\lambda k_{B}T}\right)-1}\;\left[W\cdot m^{-2}\cdot sr^{-1}\cdot \mu m^{-1}\right]\;$$

where h is Planck’s constant, c is the speed of light, and k is Boltzmann’s constant.

**Figure 4:**
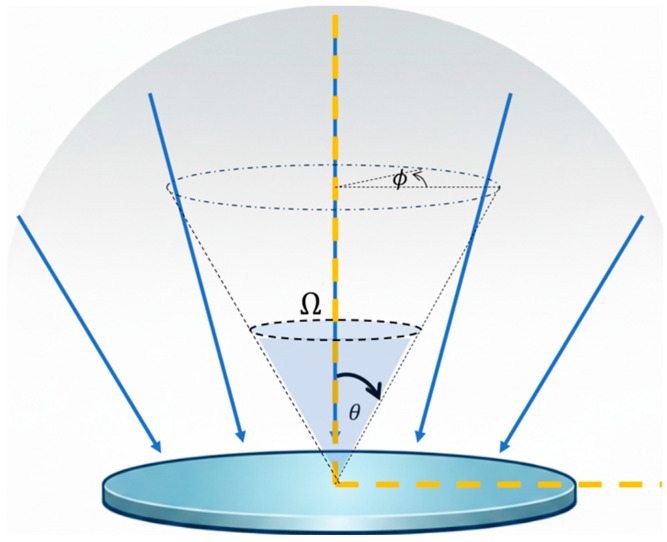
Collection cone of the TMOS sensor’s optical system. Incident radiation arrives within a solid angle defined by polar and azimuthal angles, with spectral irradiance.

The spectral irradiance, $E_{\lambda }$, at the sensor plane is obtained by integrating radiance over the collection cone of the optical system:

(2) $$E_{\lambda }\left(T\right)=\int_{\Omega }L_{\lambda }\left(T\right)\;cos\theta \;d\Omega \;\left[W\cdot m^{-2}\cdot \mu m^{-1}\right]$$

where Ω is the solid angle defined by the numerical aperture of the optics and T is the object temperature. The factor cosθ in the irradiance equation accounts for the angle of incidence relative to the sensor’s normal axis, where θ, is the polar angle, shown in Figure 4.

The total absorbed spectral power in a detector pixel of effective area $A_{\mathrm{eff}}$ is then

(3) $$P_{\mathrm{abs}}\left(\lambda \right)=A_{\mathrm{eff}}\;\eta_{\mathrm{abs}}\left(\lambda \right)\;E_{\lambda }\left(T\right)\;\;\left[W\cdot \mu m^{-1}\right]$$

where $\eta_{\mathrm{abs}}(\lambda )$ is the absorption efficiency obtained from the FDTD simulations.

In practice, the FDTD results were normalized to unit incident irradiance (1 W·m^−2^). Using the above relations, the normalized absorption spectra (optical efficiencies) were scaled to the blackbody-derived irradiance corresponding to a given source temperature and optical collection geometry. The resulting absorbed power distribution was used as the volumetric heat generation input for the thermal FEA (Section 4).

### 3.2. Power Absorbed in a Dispersive and Absorptive Material

To couple the optical and thermal domains, the volumetric heat generation in the Metasurface absorber is computed from the local electromagnetic dissipation. Starting from Poynting’s theorem, the power dissipated per unit volume in a dispersive and absorptive medium is expressed as the time-averaged product of the electric and magnetic fields with the imaginary part of the material’s permittivity and permeability [26,27,28]:

(4) q=12ω ε0 Imεω∣E∣2+12ω μ0 Imμω∣H∣2W·m−3

where ω is the angular frequency of the incident light, E and H are the local electric and magnetic fields, and ε(ω) and μ(ω) are the complex, frequency-dependent permittivity and permeability of the material. For non-magnetic materials (μ(ω)≈1), the magnetic contribution is negligible, yielding q=12ω ε0 Im{ε(ω)}∣E∣2.

In this work, the three-dimensional spatial distribution of ⟨q(r)⟩ is computed using full-wave FDTD simulations of the Metasurface absorber. The resulting volumetric heat generation profile is exported to the thermal finite element model and applied as a distributed source term for subsequent thermal analysis (Section 4). This approach ensures a physically consistent mapping from electromagnetic absorption to the thermal response of the CMOS-SOI-MEMS pixel.

### 3.3. Optical Efficiency Analysis

The optical performance of the MEMS thermal IR sensor is dictated by the efficiency with which the suspended CMOS membrane absorbs incident infrared radiation. Since the absorbed power is directly converted into heat and subsequently sensed as a temperature rise, maximizing absorption is a critical design goal. In this study, two absorption-enhancement strategies are investigated. First, we examine the intrinsic absorption of the CMOS device layer, both in its unmodified form and with an embedded titanium nitride (TiN) layer that improves optical-to-thermal conversion. Second, we evaluate advanced metamaterial absorber designs based on metal–insulator–metal (MIM) stacks, which further tailor the spectral response and boost efficiency. The following subsections present the modeling framework and simulation results for these approaches.

#### 3.3.1. CMOS Layers Absorption and TiN-Embedded Layer

**Figure 5:**
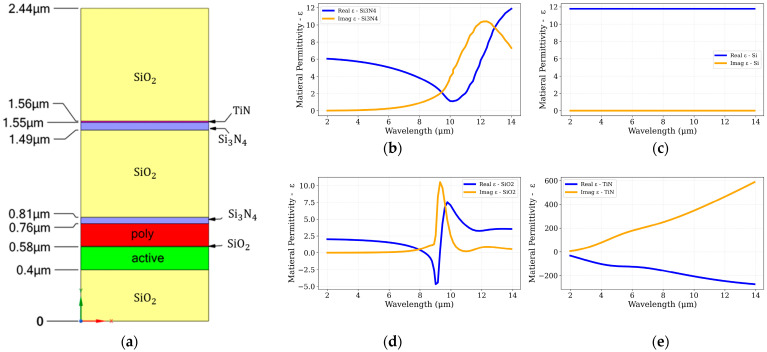
(**a**) Cross-sectional schematic of the CMOS-SOI multilayer stack used in the optical simulations, including Si, SiO_2_, Si_3_N_4_, and an embedded TiN layer. (**b**–**e**) Wavelength-dependent complex permittivity, ε(λ)=Re(ε)+i·Im(ε), for the materials comprising the stack: (**b**) silicon nitride (Si_3_N_4_), (**c**) silicon (Si), (**d**) silicon dioxide (SiO_2_), and (**e**) titanium nitride (TiN). The real part of the permittivity (blue lines) indicates refractive and dispersive properties, while the imaginary part (orange lines) highlights absorption characteristics critical to mid-infrared performance.

To accurately model the intrinsic absorption of the CMOS device layer, it is necessary to account for the layered structure of the membrane and the optical properties of its constituent materials. Figure 5 presents the input data based on [19,29,30] that used in the FDTD simulations and the device material layers.

The cross-sectional schematic of the CMOS–SOI stack illustrates a multilayer configuration comprising alternating dielectric and conductive films. From top to bottom, the structure includes silicon dioxide (SiO_2_), an embedded titanium nitride (TiN) layer, an additional SiO_2_ layer, a thin silicon nitride (Si_3_N_4_) film, and another SiO_2_ layer. These layers are followed by the polysilicon gate and the active silicon device layer, supported on a thick buried SiO_2_ layer.

The complex permittivity spectra highlight the distinct optical characteristics of each constituent material. Silicon remains nearly lossless across the 2–14 μm spectral range, ensuring transparency in the mid-infrared region. SiO_2_ and Si_3_N_4_ exhibit pronounced phonon-polariton resonances beyond 8–9 μm, resulting in wavelength-selective absorption peaks. In contrast, TiN demonstrates metallic behavior, with a negative real permittivity and an increasing imaginary component, enabling efficient broadband light absorption.

Beyond its intrinsic optical loss, the TiN layer can also contribute to enhanced absorption by acting as an optical cavity within the dielectric stack. Its strategic placement and thickness strongly influence interference and field confinement effects, thereby modifying the local absorption distribution. The location and thickness of the TiN layer were systematically studied to optimize its contribution to the overall optical performance z [31].

These combined material and structural properties govern how the CMOS–SOI stack interacts with incident infrared radiation. The embedded TiN layer enhances absorption efficiency within the mid-IR band by introducing tunable loss and resonant field confinement, while remaining fully compatible with standard CMOS fabrication processes.

**Figure 6:**
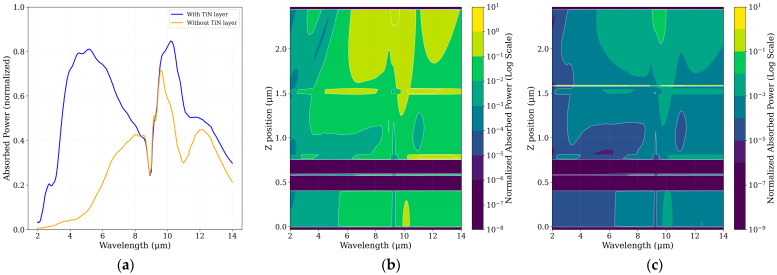
Role of layer in mid-infrared absorption enhancement and confinement. (**a**) Simulated normalized absorbed power versus wavelength. The inclusion of the embedded TiN (blue curve) yields a substantial and broadband enhancement of absorption across the entire mid-infrared spectrum, greatly surpassing the performance of the structure without the layer (orange curve). (**b**,**c**) Normalized absorbed power maps (Log Scale) across the detector cross-section, without (**b**) and with (**c**) the layer. Notably, the silicon substrate (deep blue/purple regions) exhibits negligible absorption, confirming its optical transparency in the studied IR range and the necessity of highly absorptive elements in the stage layers. The TiN layer in (**c**) is shown to induce strong field confinement and localized absorption, primarily within the active dielectric stack, validating its role as an effective, integrated light-trapping medium to compensate for the non-absorbing substrate.

FDTD simulations were conducted using Lumerical FDTD [19] to quantify the absorption efficiency of the CMOS–SOI sensor stack over the 2–14 µm range. Two configurations were analyzed: the baseline CMOS stack without TiN and the same structure incorporating a 100 nm embedded TiN layer. The absorption efficiency, defined as the ratio of absorbed to incident optical power, was used to directly compare both designs, as shown in Figure 6.

#### 3.3.2. Metamaterial Absorber Layers

While the embedded TiN layer enhances broadband absorption, further improvement in both efficiency and spectral selectivity can be achieved through the implementation of engineered metamaterial absorbers. These structures, based on metal–insulator–metal (MIM) stacks, are designed to support wavelength-selective resonant cavity modes that confine and trap infrared radiation within the sensing platform. By tailoring the geometric and material parameters, MIM absorbers can be engineered to operate as narrowband band-pass filters, selectively enhancing absorption at specific wavelengths of interest.

In this study, two representative MIM configurations—denoted MIM 1 and MIM 2 were designed and analyzed, each differing in layer thickness and overall stack geometry. The bottom metallic layer is optically opaque, ensuring zero transmission and allowing the simulation of a single unit cell. To emulate an extended Metasurface array integrated on top of the CMOS–SOI–MEMS sensor pixel, periodic boundary conditions were applied along the lateral directions, while perfectly matched layer (PML) boundaries were used along the top and bottom to eliminate spurious reflections.

**Figure 7:**
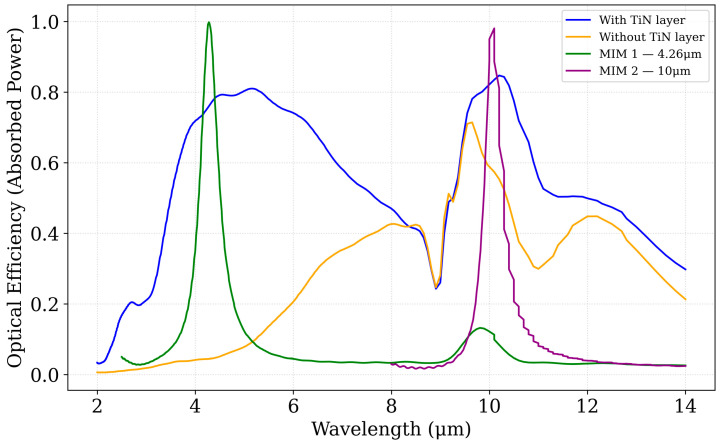
Simulated spectral optical efficiency response showing the normalized absorbed power as a function of wavelength.

Simulation results demonstrate that both MIM designs substantially enhance optical absorption within the targeted mid- and long-wavelength infrared regions, exhibiting near-unity efficiency at their respective resonance wavelengths. These resonant peaks, corresponding to the designed cavity modes, confirm the tunable narrowband filtering behavior of the MIM structures. The resulting absorption spectra for the baseline CMOS sensor, the CMOS + TiN stack, and the two MIM-enhanced configurations are presented in Figure 7.

From Figure 7, it is evident that incorporating the MIM1 structure significantly enhances the optical efficiency at ~10 µm, achieving nearly perfect absorption with an efficiency of 0.99. In contrast, without the MIM1 layers, the CMOS stack yields only 0.7564 when including the TiN layer and as low as 0.032 in the absence of TiN. Likewise, the addition of MIM2 at ~10 µm improves the efficiency to 0.9804, compared to 0.8353 for the CMOS–TiN configuration without MIM2 and 0.5740 when TiN is not present.

**Figure 8:**
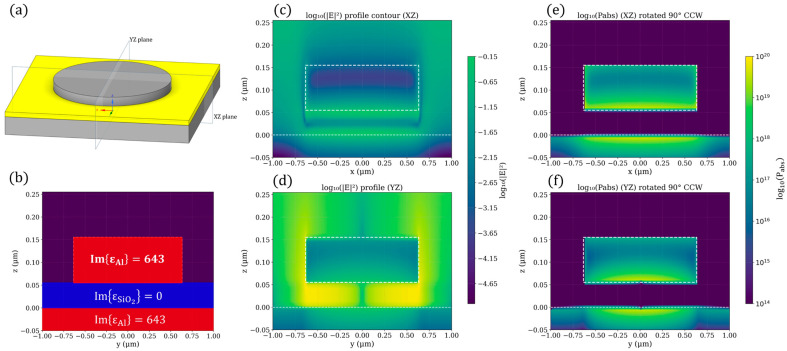
Optical response and field distributions of the MIM Metasurface at the peak absorption wavelength of 4.26 µm. (**a**) Three-dimensional schematic of the MIM unit cell, indicating the cross-sectional XZ and YZ planes used for field analysis. (**b**) Cross-sectional distribution of the imaginary part of the permittivity in the YZ plane, showing the layered configuration comprising the resonator (**top**), spacer (**middle**), and ground plane (**bottom**). (**c**,**d**) Electric field intensity distributions in the XZ (**c**) and YZ (**d**) planes, demonstrating strong field confinement within the resonant cavity. (**e**,**f**) Absorbed optical power distributions in the XZ (**e**) and YZ (**f**) planes, illustrating regions of maximum photon-to-heat conversion localized mainly within the metallic layers.

Furthermore, electric field and absorbed power density distributions were computed to illustrate how light is confined and absorbed within the MIM layers (see Figure 8), providing insight into the mechanisms of enhanced absorption. These results demonstrate the potential of MIM configuration to substantially improve the photon-to-heat conversion efficiency of CMOS-SOI-MEMS infrared sensors.

The strong absorption originates from resonant coupling between the incident wave and cavity modes confined within the MIM stack. The top patterned metal and the bottom ground plane form a Fabry–Perot–like cavity, where destructive interference suppresses reflection and traps energy inside the dielectric spacer. The confined fields are strongly enhanced at the metal–dielectric interfaces, leading to ohmic losses and efficient photon-to-heat conversion within the metallic layers (Figure 8). Such impedance-matched plasmonic resonances are known to govern selective absorption in MIM Metasurfaces [32,33].

**Figure 9:**
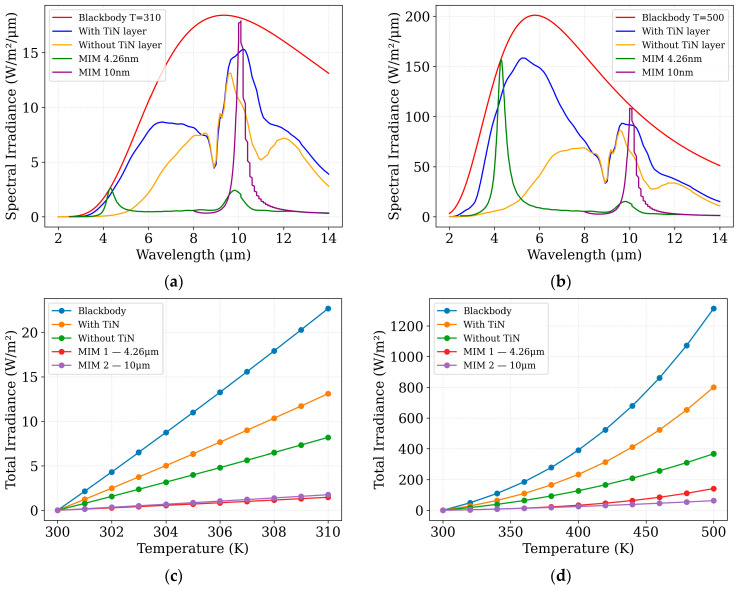
Schematic Spectral and total irradiance incident on the sensor for different object temperatures. Panels (**a**,**b**) show the spectral irradiance for objects at 310 K and 500 K, respectively, while panels (**c**,**d**) present the total integrated irradiance for temperature ranges of 300–310 K and 300–500 K. These results highlight how both spectral content and total power incident on the sensor vary with object temperature.

Using the simulated optical absorption efficiency as a function of wavelength, the spectral and total irradiance incident on the sensor from an object at temperature T can be calculated. To illustrate the effect of object temperature on the irradiance received by the sensor, Figure 9 presents both the spectral and integrated irradiance for two representative temperature ranges: 300–310 K and 300–500 K. These plots provide insight into the amount of optical power delivered to the sensor under different thermal conditions, which is critical for evaluating the sensor’s response.

Using the total irradiance, the total absorbed power (Equation (3)) can be computed and subsequently applied as a heat source in the thermal simulations (Section 4). This approach enables the evaluation of the thermal impact of the metamaterial layers on the performance of the CMOS-SOI-MEMS sensor pixel.

## 4. Thermal Analysis

The thermal performance of the TMOS sensor determines its responsivity to incident IR radiation. This section presents a thermal modeling framework that quantifies temperature rise (ΔT) and dynamic response, integrating optical heat sources from FDTD simulations (Section 3). The framework employs FEA and an analytical lumped-element RC circuit model to evaluate the impact of Metasurface absorbers on thermal behavior. Applicable to any absorber architecture—narrowband, broadband, or multilayer—the framework ensures versatility for diverse IR applications.

### 4.1. Governing Principles of Heat Transfer

The thermal behavior of MEMS IR sensors is dominated by conduction through the suspended support beams and solid layers, with radiation as a secondary mechanism. Convection is negligible in vacuum. The transient heat diffusion equation is [34,35]

(5a) $$\rho c_{p}\frac{\partial T}{\partial t}=k\nabla^{2}T+Q_{gen}$$

(5b) $$\frac{\partial T}{\partial t}=\alpha \nabla^{2}T+\frac{Q_{gen}}{\rho c_{p}}$$

where ρ is density, $c_{p}\;\left[J\cdot kg^{-1}\cdot K^{-1}\right]$ is specific heat capacity, $k\;\left[W\cdot m^{-2}\cdot K^{-1}\right]$ is thermal conductivity, T is temperature, α is the thermal diffusivity and defined as $\alpha \equiv \frac{k}{\rho c_{p}}\;\left[m^{2}\cdot s^{-1}\right]$ and $Q_{gen}\;\left[W\cdot m^{-3}\right]$ is the volumetric heat source.

### 4.2. Lumped-Circuit Equivalent Model

For microscale devices, when the thermal diffusion length $L_{d}=\sqrt{\alpha \tau_{th}}$ exceeds the platform size, the stage temperature can be assumed uniform. The sensor can then be modeled as a lumped system:

(6) $$L_{d}=\sqrt{\alpha \tau_{th}}\;\left[m\right]$$

If $L_{d}$ exceeds the dimensions of the micro-platform, temperature gradients within the stage are negligible, allowing the entire platform to be modeled as a lumped thermal capacitance $C_{\mathrm{th}}$ connected to the substrate through an overall thermal conductance $G_{\mathrm{th}}$ [16,36]:

(7) $$C_{\mathrm{th}}\frac{d\Delta T(t)}{dt}+G_{\mathrm{th}}\Delta T(t)=P_{\mathrm{opt}}(t)$$

where ΔT is the temperature rise of the sensing stage and $P_{\mathrm{opt}}\;\left[W\right]$ is the absorbed optical power. The thermal conductance of a single support beam is obtained from Fourier’s law:

(8) $$G_{\mathrm{th}}=\frac{kA}{L}$$

where A is the beam cross-sectional area, and L its length. For multilayer or heterogeneous beams structures, equivalent thermal conductivities can be computed for series or parallel arrangements, similar to electric conductivity [16,37,38]:

(9) $$k_{\mathrm{eq},\mathrm{serial}}=L{\left(\sum_{i=1}^{n}\frac{l_{i}}{k_{i}}\right)}^{-1},\;k_{\mathrm{eq},\mathrm{parallel}}=\frac{1}{A}\sum_{i=1}^{n}k_{i}A_{i}$$

with $l_{i},k_{i},A_{i}$ as the length, conductivity, and cross-section of each material.

The total thermal capacitance is obtained by summing contributions from all constituent materials:

(10) $$C_{\mathrm{th}}=\sum_{i=1}^{n}\rho_{i}c_{i}V_{i}$$

where $V_{i}$ is the volume of the i-th layer. Equivalently the thermal time constant is defined by $\tau_{th}=C_{th}/G_{th}$.

The thermal and physical properties of the materials relevant to the CMOS–SOI stack are summarized in Table 1.

**Figure 10:**
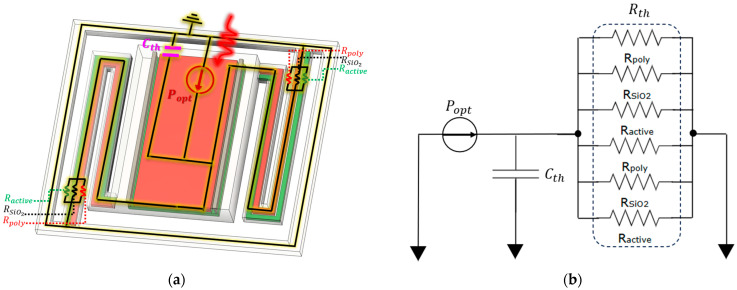
Illustration of the lumped-element thermal model for the CMOS-SOI-MEMS sensor pixel: (**a**) 3D schematic of the sensor pixel with the stage and supporting arms, and (**b**) equivalent thermal circuit representation, where the stage is modeled as the dominant thermal capacitance and the arms as the primary thermal resistors.

The material parameters summarized in Table 1 correspond to a reference temperature of 25 °C. As shown in the thermal simulations, the sensor’s temperature rises due to absorbed infrared radiation is on the order of only a few millikelvin (mK). Therefore, for the present analysis, these small variations are assumed not to affect the thermal properties. However, for operation under different ambient conditions or elevated temperatures, the temperature dependence of the thermal parameters (such as thermal conductivity, specific heat, and density) should be considered, as they may introduce measurable deviations in the thermal response. The sensor pixel is modeled under the lumped thermal assumption, where the suspended stage and MOSFET layers (with or without MIM additions) constitute the dominant thermal capacitance, and the supporting arms act as the primary thermal resistors. Heat flows independently through each arm, forming parallel thermal paths that reduce the overall thermal resistance. Figure 10 depicts both the 3D pixel structure and its equivalent thermal circuit.

### 4.3. Simulation Setup and Results

The 3D FEA model was constructed using the material properties listed in Table 1. The frame-connected edges of the supporting arms were treated as isothermal boundaries, representing the bulk silicon heat sink. Heat input was applied to the sensor stage, corresponding to the absorbed optical power calculated from the optical efficiency and irradiance analysis (Section 3), defined as the difference between incident irradiance from the target and the sensor’s radiative emission at its initial temperature:

(11) $$P_{\mathrm{opt}}=\int_{\lambda_{\min}}^{\lambda_{\max}}P_{\mathrm{abs}}\;\left(\lambda \right)d\lambda -\int_{\lambda_{\min}}^{\lambda_{\max}}P_{emitted}\left(\lambda \right)d\lambda =\int_{\lambda_{\min}}^{\lambda_{\max}}A_{\mathrm{eff}}\;\eta_{\mathrm{abs}}\left(\lambda \right)E_{\lambda }\left(T_{target}\right)d\lambda -\int_{\lambda_{\min}}^{\lambda_{\max}}A_{\mathrm{eff}}\;\eta_{\mathrm{abs}}\left(\lambda \right)E_{\lambda }\left(T_{0}\right)d\lambda$$

where $T_{0}$ is the initial sensor temperature and $T_{\mathrm{target}}$ is the temperature of the observed object that emits the incident radiation.

**Figure 11:**
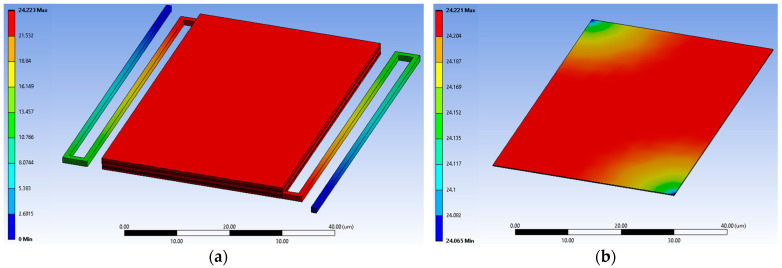
Steady-state temperature rise of the CMOS-SOI-MEMS sensor pixel without MIM layers: (**a**) Full pixel, illustrating heat dissipation through the supporting arms. (**b**) Active silicon layer of the stage, highlighting the thermal uniformity. The very close limits on the color bar (Min: 24.065, Max: 24.221 mK) prove the temperature distribution is approximately uniform across the sensing area.

Given the lumped thermal assumption and the large thermal diffusion length relative to the stage dimensions, the detailed spatial distribution of absorbed power is not critical. Although FDTD simulations provide fine-scale absorption profiles, their mesh cannot be directly matched to the coarser FEA thermal mesh. Therefore, the total absorbed optical power $P_{opt}$ is applied uniformly across the sensor stage, yielding comparable thermal results with significantly reduced computational complexity. Figure 11 illustrates the steady-state temperature distribution for a target object temperature of 301 K, assuming an initial sensor temperature of 300 K, for the CMOS-SOI-MEMS sensor with the TiN layer and without MIM structures, validating the lumped model assumption.

**Figure 12:**
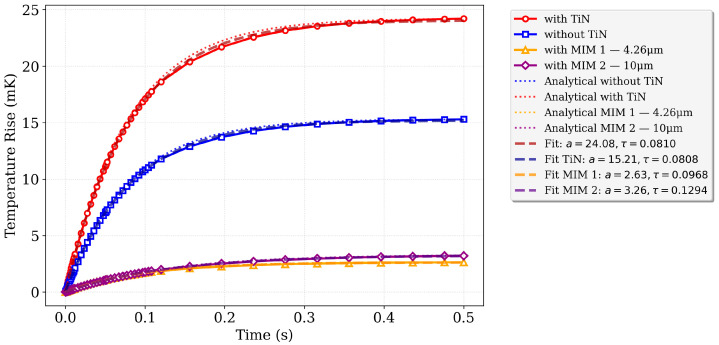
Transient thermal response (ΔT(t)) of the sensor stage for different material configurations: without TiN, with TiN, with TiN and MIM configuration 1, and with TiN and MIM configuration 2. Analytical predictions from the lumped-element thermal model are included for comparison, illustrating the effects of TiN and MIM layers on the heating dynamics and validating the lumped thermal modeling approach.

As shown in Figure 11, the stage and active MOSFET layer exhibit a nearly uniform temperature, while significant gradients occur along the support arms, validating the lumped thermal model. To assess the dynamic thermal behavior, the transient stage temperature ΔT(t) was simulated for four configurations: without TiN, with TiN, with TiN and MIM configuration 1, and with TiN and MIM configuration 2. Figure 12 presents the transient results of these simulations, alongside predictions from the analytical lumped-element model, enabling direct comparison with the full 3D FEA results. Thermal capacitance and conductance were computed from Equations (7)–(11) using the material parameters in Table 1, and the FEA simulations were fitted to both the analytical solution and the corresponding $G_{th}$ and $C_{th}$ values.

Figure 12 demonstrates excellent agreement between the full 3D FEA results and the analytical thermal circuit model. For the sensor without TiN, the analytical time constant is 78.65 ms, closely matching the FEA value of 81 ms. With TiN, the values are 78.93 ms (analytical) and 80.80 ms (FEA). Incorporation of MIM layers increases thermal capacitance and the corresponding time constant: MIM configuration 1 yields 94.83 ms (analytical) versus 96.8 ms (FEA), and MIM configuration 2 yields 127.50 ms (analytical) versus 129.4 ms (FEA), confirming the model’s predictive accuracy.

## 5. Mechanical Analysis

The dynamic mechanical integrity of MEMS is a fundamental determinant of sensor performance, stability, and reliability [39]. Specifically, the natural frequencies and mode shapes dictate the susceptibility response to external vibrations, which can cause excessive displacement, structural fatigue, or noise-inducing signal distortion. This analysis verifies that the operational bandwidth is safely decoupled from the device’s mechanical resonances.

### 5.1. Modal and Harmonic Analysis

**Figure 13:**
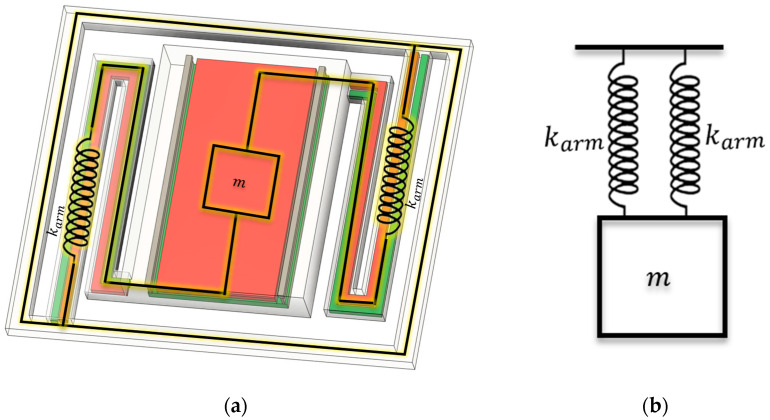
Mechanical modeling of the suspended CMOS sensor. (**a**) Three-dimensional rendering of the MEMS pixel showing the suspended thermal mass and the supporting arms that provide the effective spring stiffness. (**b**) The equivalent single-degree-of-freedom mass-spring system, which simplifies the dynamic analysis and provides intuitive insight into the natural frequency.

The dynamic behavior of the suspended micro-platform is often conceptually modeled as a mass-spring system to provide intuitive insight, with the equivalent system illustrated in Figure 13. For the undamped, single-degree-of-freedom approximation, the governing equation is [38,40]

(12) $$m\ddot{x}(t)+k_{m}x(t)=0$$

with a harmonic solution $x(t)=Xe^{j2\pi f_{n}t}$ yielding the natural frequency:

(13) $$f_{n}=\frac{1}{2\pi }\sqrt{\frac{k_{m}}{m}}$$

For the complex, multi-material 3D MEMS microstructure a FEM eigenvalue problem is necessary to capture all vibration modes:

(14) $$\left[K]\{\phi \}=\omega_{n}^{2}[M]\{\phi \right\}$$

where [K] and [M] are the global stiffness and mass matrices, $\omega_{n}$ are the natural angular frequencies, and {ϕ} are the corresponding mode shapes. Each mode represents a distinct three-dimensional deformation of the suspended platform.

To assemble the stiffness [K] and mass [M] matrices, the FEM solver requires the material properties of each structural component: density ρ, Young’s modulus E, and Poisson’s ratio ν. These parameters define the inertial and elastic response of the microstructure. The mechanical properties are depicted in Table 2.

**Figure 14:**
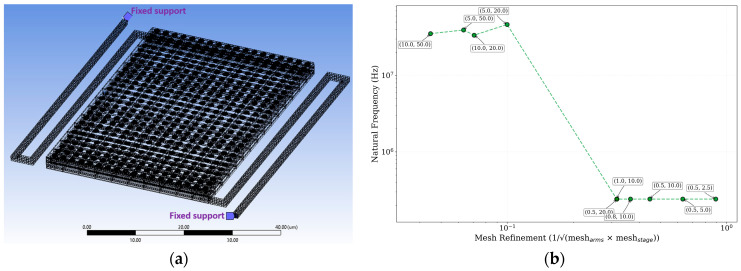
FEA model of the suspended microbolometer pixel. (**a**) The 3D geometry is discretized using a dense mesh for accurate mechanical simulation. Fixed support boundary conditions are applied at the anchor points where the suspending arms connect to the rigid sensor frame, simulating the physical constraints on the device. (**b**) Mesh convergence analysis showing natural frequency vs. mesh refinement parameter $\left(1/\sqrt{mesh_{arms}\times \;mesh_{stage}}\right)$. Data points are labeled with (arms mesh size, stage mesh size) values. The study demonstrates convergence at mesh refinement values ≥ 0.4 µm^−1^, corresponding to arms mesh ≤ 1.0 µm.

The FEA model was constructed with a dense mesh for discretization, and fixed support boundary conditions were applied at the anchor points (Figure 14), consistent with the methodology used for similar high-performance MEMS devices. This simulates the physical constraint where the suspending arms connect to the rigid substrate frame.

The harmonic response analysis quantifies the steady-state displacement when the MEMS device is subjected to a sinusoidal external force $F(t)=F_{0}e^{j\omega_{m}t}$, the general equation of motion for a single-degree-of-freedom system is

(15) $$m\ddot{x}(t)+c\dot{x}(t)+k_{m}x(t)=F_{0}e^{j\omega_{m}t}$$

with the steady-state displacement amplitude:

(16) $$|X(\omega )|=\frac{F_{0}}{\sqrt{(k_{m}-m\omega_{m}^{2}{)}^{2}+(c_{m}\omega {)}^{2}}}$$

where $c_{m}$ accounts for damping due to air, anchors, and internal dissipation. This frequency response function highlights resonance peaks near the natural frequencies. In practice, the MEMS sensor has many degrees of freedom, and its full dynamics are captured by the finite element formulation:

(17) $$\left[M]\{\ddot{u}(t)\}+[C]\{\dot{u}(t)\}+[K]\{u(t)\}=\{F(t)\right\}$$

where {u(t)} contains all nodal displacements. This multi-DOF analysis provides the accurate three-dimensional vibration characteristics, while the lumped single-DOF model offers intuitive insight into resonance behavior. Since the TMOS sensor is wafer-level packaged (WLP) in deep vacuum (a few pascals), the damping coefficients for MEMS devices are reported to be in the range of 0.0005–0.002 [41]. Therefore, in our harmonic response analysis we used the mean value of this range, 0.00125. Because these damping values are very small, the quality factor (Q) is very high and approximately equal for this entire range, and the peak resonance frequency may vary by only a few kilohertz across this range of damping coefficients.

Residual mechanical stress inevitably develops within multilayer CMOS–MEMS sensor stacks due to differences in material properties and fabrication thermal history. These stresses originate from both intrinsic sources (e.g., film growth mechanisms, ion bombardment during deposition) and extrinsic sources such as thermal expansion mismatch between adjacent layers during cooling from deposition or annealing. The resulting stress can lead to out-of-plane deformation, membrane curvature, or even delamination, ultimately affecting the optical alignment and long-term reliability of the sensor.

The thermally induced biaxial stress in a thin film deposited on a substrate can be expressed as [38]

(18) $$\sigma =\frac{E_{f}}{1-\nu_{f}}\left(\alpha_{f}-\alpha_{s}\right)\Delta T$$

where $E_{f}$ and $\nu_{f}$ are the Young’s modulus and Poission’s ratio of film, $\alpha_{f}$ and $\alpha_{s}$ are coefficients of thermal expansions of the film and substrate, and ΔT is the temperature change between deposition and operation. Tensile stress occurs if $\alpha_{f}>\alpha_{s}$ during cooling, while compressive stress occurs if $\alpha_{f}<\alpha_{s}$.

### 5.2. Mechanical Simulation Results and Physical Interpretation

The vibrational characteristics were investigated across three critical configurations: the baseline structure (Without MIM), and two configurations integrating the MIM absorber layer (With MIM 1 and With MIM 2). The addition of the absorber introduces mass loading that directly modifies the effective stiffness–mass balance, $\sqrt{k_{m}/m}$.

Table 3 summarizes the first six natural frequencies. A clear downward shift in the lower-order frequencies is observed with the inclusion and thickening of the MIM absorber layer.

**Figure 15:**
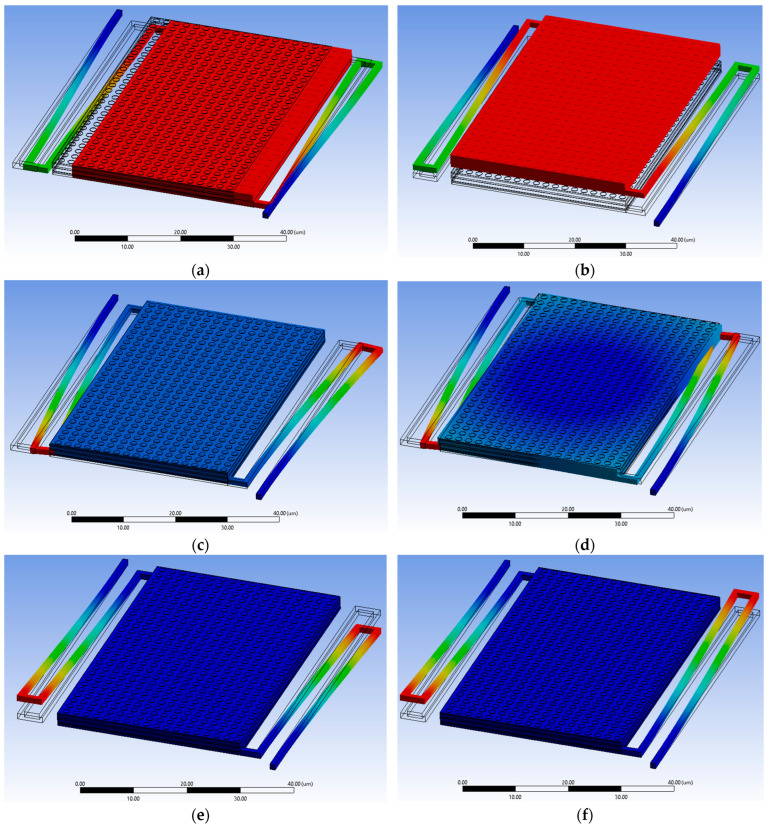
First six vibration mode shapes of the MEMS IR sensor: (**a**,**b**) first and second modes showing out-of-plane and torsional motion of the suspended stage, (**c**,**d**) third and fourth modes with combined stage and slight arm deformation, and (**e**,**f**) fifth and sixth modes dominated by arm bending and twisting. The color scale represents displacement magnitude, with red indicating maximum displacement and blue indicating minimum displacement (fixed anchors). Note that the absolute displacement values are arbitrary and serve only to visualize the mode shapes; the modal analysis extracts the natural frequencies and relative deformation patterns under the assumption of linear elastic behavior and small displacements.

Figure 15 illustrates the corresponding mode shapes, revealing a critical distinction in the dynamic response:

- Low-Order Modes ($f_{1}$ to $f_{4}$): Stage-Dominated Motion. These modes exhibit out-of-plane translation, torsional rotation, and combined deformation of the suspended platform itself (Figure 15a–d). The substantial frequency reduction (e.g., dropping from 31.03 kHz to 24.88 kHz with MIM 2) is a direct consequence of the MIM’s mass being concentrated on the suspended stage.

- High-Order Modes ($f_{5}$ and $f_{6}$): Arm-Dominated Motion. These modes, occurring at significantly higher frequencies (), primarily involve the bending and twisting of the support arms (Figure 15e,f). Crucially, their frequencies are negligibly affected by the MIM layers. This confirms that the MIM integration selectively affects the stage’s inertial properties while preserving the high stiffness and mechanical integrity of the support structure. The high arm frequencies (the fundamental frequency) are essential for ensuring thermal isolation compliance without introducing low-frequency structural vulnerabilities.

**Figure 16:**
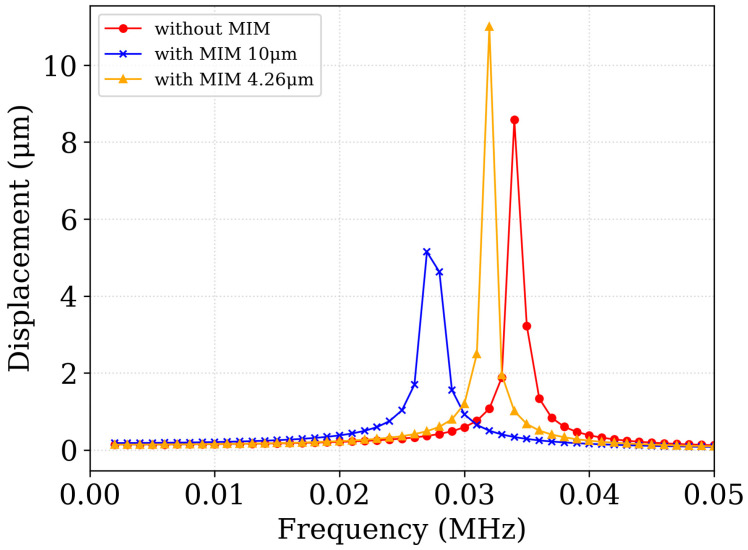
Harmonic displacement response of the MEMS thermal IR sensor for three configurations: without MIM, with MIM 1, and with MIM 2. The resonance peaks align with the modal frequencies reported in Table 3, showing the expected downward frequency shift with increasing MIM thickness and a moderate reduction in peak amplitude due to the added mass loading.

The harmonic response analysis was then performed to evaluate the displacement amplitude of the suspended structure under sinusoidal excitation. Figure 16 shows the frequency response for the three configurations. The resonance peaks correspond closely to the modal frequencies listed in Table 2, confirming the consistency of the two approaches.

The simulation results confirm that the sensor’s operational bandwidth lies well below its fundamental resonance frequency, ensuring that normal operation remains unaffected by mechanical resonance effects. This verifies that the suspended stage maintains dynamic stability under typical excitation conditions.

Critically, the lowest natural frequency of the sensor with the MIM absorber is approximately 28 kHz, which is substantially higher than the frequency range of common environmental disturbances encountered in both indoor and outdoor settings (typically below 1 kHz). This indicates strong mechanical robustness, with minimal susceptibility to destructive vibrations or excessive noise coupling during operation.

Furthermore, while the metamaterial MIM layers slightly reduce the resonance frequencies due to their added mass, they do not significantly compromise the overall mechanical stability of the device. The modest frequency shift demonstrates that the MIM layers can be integrated without adversely affecting the structural integrity of the suspended platform. Thus, the metamaterial absorber enhances optical absorption performance while preserving the sensor’s mechanical characteristics.

By maintaining sufficiently high natural frequencies, the design ensures that environmental vibrations and mechanical impacts do not interfere with thermal signal detection. This provides confidence that the MEMS IR sensor can operate reliably in real-world deployment scenarios without mechanical resonance degrading sensitivity or stability.

**Figure 17:**
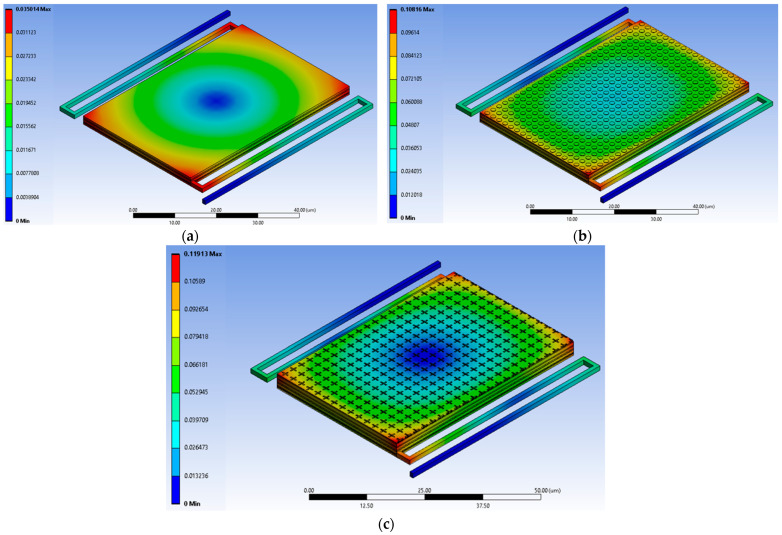
Simulated total deformation of the sensor structure during cooldown from a metal deposition temperature of 400 °C (ΔT = –400 °C), using the coefficients of thermal expansion from Table 2. (**a**) Sensor without MIM absorber, showing smooth deformation with a maximum of 0.035 µm. (**b**) Sensor with MIM 1 absorber, exhibiting a maximum deformation of 0.10816 µm. (**c**) Sensor with MIM 2 absorber resulting in a maximum deformation of 0.11913 µm. All scale bars are in micrometers (µm).

To evaluate the impact of residual thermal stress on device deformation, we simulated the sensor cooldown and MEMS release process. Assuming typical metal deposition temperatures of approximately 400 °C, a thermal change of ΔT = −400 °C was applied using the coefficients of thermal expansion listed in Table 2. The resulting total deformation of the sensor, with and without the metal–insulator–metal (MIM) absorber configuration, is shown in Figure 17.

The simulation results clearly indicate that incorporating the MIM absorber significantly increases the total device deformation. This increase is directly attributable to the higher coefficient of thermal expansion α of the additional aluminum metal layers compared to the Silicon-Dioxide and silicon layers, leading to greater thermal stress and out-of-plane bending during cooldown. However, despite this increase, the maximum deformation remains relatively small at approximately 0.1 µm. This small magnitude, coupled with the likely absence of steep deformation gradients across the active sensor area, suggests that the flatness and structural integrity of the sensor’s sensing surface are largely maintained. Therefore, the overall performance of the sensor is anticipated to be superior with the MIM configuration due to the substantial gain in absorption efficiency outweighing the negligible impact of the small residual thermal stress-induced deformation.

## 6. Discussion

This study develops a comprehensive multiphysics modeling framework that integrates optical, thermal, and mechanical analyses to optimize CMOS-SOI-MEMS TMOS sensors with Metasurface absorbers. The following subsections synthesize the results, elucidate trade-offs, and highlight the framework’s utility for rapid design optimization, particularly through the efficient thermal analytical model.

FDTD simulations demonstrate that Metasurface absorbers achieve near-unity absorption at targeted wavelengths (e.g., 0.99 at 4.26 µm for MIM 1, compared to 0.7564 with TiN layer and 0.032 without) (Section 3.3.2). The TiN layer enhances broadband absorption across 2–14 µm, while MIM configurations enable spectral selectivity for applications like CO_2_ sensing (4.26 µm) and thermal imaging (10 µm). The optical modeling framework’s versatility, applicable to any absorber architecture-narrowband, broadband, multilayer, or varied geometries-ensures broad utility for tailored IR sensor designs.

Thermal FEA confirms the TMOS sensor’s high thermal isolation, maximizing temperature rise (ΔT) for a given IR flux (Section 4). Metasurface integration, however, increases thermal capacitance, extending the thermal time constant from 80 ms (TiN) to 95 ms (MIM 1) and 129 ms (MIM 2). While suitable for optical gas sensing, this may limit temporal bandwidth in fast-detection scenarios, necessitating careful absorber optimization. A key advancement is the analytical lumped-element RC circuit model, which exhibits excellent agreement with 3D FEA (e.g., 78.93 ms vs. 80.80 ms for TiN) (Section 4.3). This model enables rapid evaluation of diverse layer and Metasurface absorber structures—narrowband, broadband, or multilayer—without the computational overhead of full 3D FEA simulations, which are significantly more resource-intensive [34]. Mechanically, modal and harmonic analyses verify structural resilience, with natural frequencies remaining above 20 kHz despite Metasurface mass loading (e.g., 31.03 kHz to 24.88 kHz for MIM 2) (Section 5.2), ensuring stability against environmental vibrations (<1 kHz).

A recent study [42] presents a Multiphysics approach integrating optical and thermal modeling to enhance gas sensor performance using toroidal dipole resonances on flexible substrates. While both works share the goal of optimizing IR sensor design through advanced simulation, our framework expands the scope by incorporating mechanical analysis alongside optical and thermal domains. This enables evaluation of structural stability and vibration resilience, which are critical for CMOS-SOI-MEMS sensor deployment in real-world environments. Furthermore, our analytical RC thermal model complements full-scale simulations by enabling rapid design iterations with minimal computational cost. Unlike the referenced study, which focuses on flexible substrates and optical switching, our work targets CMOS-compatible Metasurface absorbers for thermal IR sensing, offering broader applicability across gas sensing, biomedical diagnostics, and imaging. This comparison highlights the added value of our integrated framework in addressing both performance and manufacturability.

This integrated framework advances beyond isolated domain analyses, providing a predictive tool to navigate trade-offs in absorption efficiency, thermal response, and mechanical stability. The RC circuit model’s computational efficiency facilitates rapid design iterations, enabling optimization of any absorber architecture for CMOS-compatible IR sensors. This versatility supports applications from gas sensing to biomedical diagnostics. Future work will focus on experimental validation with fabricated prototypes to refine model accuracy and explore control strategies to mitigate ambient temperature drifts, further enhancing manufacturability and performance.

While the simulated Metasurface absorber geometries presented in this study are based on CMOS-compatible materials and follow designs reported in the literature, we acknowledge that actual fabrication feasibility—particularly layer thicknesses and tolerances must be validated against the specific Process Design Kit (PDK) and in collaboration with fabrication engineers. As this work focuses on the modeling and analysis framework, the geometries serve as representative examples to demonstrate simulation capabilities. Once PDK constraints are available, the framework should be reapplied to ensure compatibility with fabrication processes and to refine the design accordingly. This step is essential for transitioning from simulation to manufacturable sensor prototypes.

Future work will focus on experimental validation with fabricated prototypes to refine model accuracy and explore control strategies to mitigate ambient temperature drifts, further enhancing manufacturability and performance.

## 7. Conclusions

This work has presented a comprehensive multiphysics modeling framework for CMOS-SOI-MEMS thermal infrared sensors integrated with Metasurface absorbers. By coupling optical, thermal, and mechanical analyses, the framework provides a predictive and quantitative approach for optimizing sensor performance across interdependent domains.

Optical simulations using the FDTD method demonstrated that Metasurface absorbers can achieve near-unity absorption at specific wavelengths, such as 4.26 µm for CO_2_ detection and 10 µm for broadband thermal imaging, while maintaining CMOS process compatibility. Thermal FEA confirmed that the TMOS structure offers excellent thermal isolation and responsivity, though Metasurface integration increases thermal capacitance and extends the time constant. The validated analytical RC model effectively reproduces full 3D FEA results, enabling rapid evaluation of absorber geometries and materials with minimal computational cost. Mechanical modal and harmonic analyses verified that the device maintains sufficient stiffness and stability, with all natural frequencies well above environmental vibration ranges.

Overall, the developed framework unifies optical absorption design, thermal performance assessment, and mechanical reliability evaluation within a single simulation environment. This approach not only accelerates design iterations but also clarifies trade-offs between responsivity, speed, and structural integrity. The presented methodology can be extended to a wide range of uncooled IR detector architectures and Metasurface configurations. Future research will focus on experimental validation through fabricated prototypes and the integration of temperature compensation and feedback mechanisms to further enhance accuracy and robustness in real-world sensing applications.

## Figures and Tables

**Table 1:** Physical and thermal properties of the sensor materials correspond to a reference temperature of 25 °C.

	Silicon	Silicon Nitride	Silicon Dioxide	Aluminum	Thin Silicon Layers and Polysilicon	Titanium Nitride
$\rho \left(kg\cdot m^{-3}\right)$	2320	3200	2200	2689	2320	5210
$k\;\left(W\cdot m^{-1}\cdot K^{-1}\right)$	140	25	1.4	237.5	40	29.1
$c\left(J\cdot {kg}^{-1}\cdot K^{-1}\right)$	700	700	730	951	678	586

**Table 2:** Mechanical properties of the sensor materials.

	Silicon	Silicon Nitride	Silicon Dioxide	Aluminum	Titanium Nitride
$E\;\left(GPa\right)$	160	250	70	70	250
ν	0.22	0.27	0.17	0.33	0.22
α (10^−6^K^−1^)	2.6	2.8	0.5	23	9

**Table 3:** First six natural frequencies of the MEMS thermal IR sensor.

	$f_{1}$ **(kHz)**	$f_{2}$ **(kHz)**	$f_{3}$ (kHz)	$f_{4}$ (kHz)	$f_{5}$ (kHz)	$f_{6}$ (kHz)
Without MIM	31.029	34.276	191.61	220.22	305.33	306.52
With MIM 1	28.87	31.89	183.79	213.17	305.35	306.38
With MIM 2	24.878	27.478	167.05	197.21	305.38	306.14

## Data Availability

Data is available upon reasonable request from the correspondence author.

## Abbreviations

The following abbreviations are used in this manuscript:

CMOS	Complementary Metal-Oxide-Semiconductor
CO_2_	Carbon Dioxide
DRIE	Deep Reactive Ion Etching
FEA	Finite Element Analysis
FDTD	Finite-Difference Time-Domain
IR	Infrared
MEMS	Micro-Electro-Mechanical Systems
MIM	Metal–Insulator–Metal
MOSFET	Metal-Oxide-Semiconductor Field-Effect Transistor
PDK	Process-Development-Kit
NDIR	Non-Dispersive Infrared
SOI	Silicon-On-Insulator
WLP	Wafer-Level Vacuum Packaging
